# Estimating the Individual Treatment Effect on Survival Time Based on Prior Knowledge and Counterfactual Prediction

**DOI:** 10.3390/e24070975

**Published:** 2022-07-14

**Authors:** Yijie Zhao, Hao Zhou, Jin Gu, Hao Ye

**Affiliations:** 1 Department of Automation, Beijing National Research Center for Information Science and Technology (BNRist), Tsinghua University, Beijing 100084, China; zhao-yj16@mails.tsinghua.edu.cn (Y.Z.); zhou17@mails.tsinghua.edu.cn (H.Z.); 2 MOE Key Laboratory for Bioinformatics, BNRIST Bioinformatics Division, Department of Automation, Tsinghua University, Beijing 100084, China; jgu@tsinghua.edu.cn

**Keywords:** individual treatment effect, survival data, counterfactual prediction, prior knowledge

## Abstract

The estimation of the Individual Treatment Effect (ITE) on survival time is an important research topic in clinics-based causal inference. Various representation learning methods have been proposed to deal with its three key problems, i.e., reducing selection bias, handling censored survival data, and avoiding balancing non-confounders. However, none of them consider all three problems in a single method. In this study, by combining the Counterfactual Survival Analysis (CSA) model and Dragonnet from the literature, we first propose a CSA–Dragonnet to deal with the three problems simultaneously. Moreover, we found that conclusions from traditional Randomized Controlled Trials (RCTs) or Retrospective Cohort Studies (RCSs) can offer valuable bound information to the counterfactual learning of ITE, which has never been used by existing ITE estimation methods. Hence, we further propose a CSA–Dragonnet with Embedded Prior Knowledge (CDNEPK) by formulating a unified expression of the prior knowledge given by RCTs or RCSs, inserting counterfactual prediction nets into CSA–Dragonnet and defining loss items based on the bounds for the ITE extracted from prior knowledge. Semi-synthetic data experiments showed that CDNEPK has superior performance. Real-world experiments indicated that CDNEPK can offer meaningful treatment advice.

## 1. Introduction

In this paper, problems related to the estimation of the Individual Treatment Effect (ITE) on survival time will be discussed. Estimating treatment effects from observational data is an important research topic of causal inference [1]. With the development of personalized healthcare, there has been increasing concern about estimating the Individual Treatment Effect on survival time, which indicates how much an individual could benefit from a pair of treatments in the sense of prolonging survival time [1], and therefore can help a doctor or a patient determine which treatment to select.

To introduce the related concepts and existing works more clearly and concisely, we need to give some notations in this section. Suppose there are N patients. For each individual patient i with baseline $x_{i}$ composed of some covariates (e.g., basic information, laboratory tests, and image tests, etc.), let $Y_{i}^{T=1}\left(x_{i}\right)$ and $Y_{i}^{T=0}\left(x_{i}\right)$ represent the potential outcomes of a pair of treatments T={1,0}. Since a patient can only receive one actual treatment of the two potential outcomes, the one which cannot be observed is referred to as counterfactual [2]. The Individual Treatment Effect of T=1 relative to T=0 is defined by$\;ITE_{i}≜\Delta_{Y_{i}}=Y_{i}^{T=1}\left(x_{i}\right)-Y_{i}^{T=0}\left(x_{i}\right)$ [1,2], which is also counterfactual because one of $\;Y_{i}^{T=1}$ and $\;Y_{i}^{T=0}$ must be counterfactual. This makes it impossible to learn a model for $\Delta_{Y_{i}}$, i.e., ${\hat{\Delta }}_{Y_{i}}\left(x_{i}\right)=f\left(x_{i}\right),$ based on historical data, which is useful for predicting the ITE for a new patient before the selecting of treatments.

Representation learning methods [1,3,4,5] are a kind of important method to deal with the counterfactual problem, which divides the historical data of all the patients into two parts, i.e., $D_{T=1}=\left\{\left(\;y_{i}^{t_{i}=1},\;x_{i}^{t_{i}=1}\right),\;i=1,...,N_{T=1}\right\}$ and $D_{T=0}=\left\{\left(\;y_{i}^{t_{i}=0},\;x_{i}^{t_{i}=0}\right),\;i=1,...,N_{T=0}\right\}$ for patients who have received treatment T=1 and T=0, respectively, with $t_{i}$ representing the actual treatment patient i has received and $y_{i}^{t_{i}=*}$ and $x_{i}^{t_{i}=*}$ representing the observed survival time and the baseline of patient i, respectively. Then, in representation learning methods, instead of learning ${\hat{\Delta }}_{Y_{i}}=f\left(x_{i}\right)$, which encounters the counterfactual problem, ${\hat{y}}_{i}^{t_{i}=1}=f_{T=1}\left(x_{i}^{t_{i}=1}\right)$ based on historical data $D_{T=1}$ and ${\hat{y}}_{i}^{t_{i}=0}=f_{T=0}\left(x_{i}^{t_{i}=0}\right)$ based on $D_{T=0}$ are learned separately, and for a new patient, the ITE can be predicted by ${\hat{\Delta }}_{Y_{new}}=f_{T=1}\left(x_{new}\right)-f_{T=0}\left(x_{new}\right)$ [3].

However, a challenge encountered by representation learning-based ITE estimation is the problem of selection bias caused by confounders, which are defined as the covariates in the baseline affecting both the treatment assignment $t_{i}\;$and the outcome$\;y_{i}^{t_{i}}$ [1,2,6]. An example [3,6] can be used to illustrate the problem. Let T=1 and T=0 denote taking a drug and not taking a drug, respectively. Suppose that most of the old patients have received treatment T=1 and most of the young patients have received treatment T=0, then in this case “age” is a confounder, which makes the data distributions of $D_{T=1}$ and $D_{T=0}$ not consistent, and therefore further leads to unreliable estimations of ${\hat{y}}_{i}^{t_{i}=0}\;$for old patients and ${\hat{y}}_{i}^{t_{i}=1}$ for young patients.

A common idea to reduce selection bias in representation learning methods for ITE estimation is balancing the confounders. As one of the typical representation learning-based methods for the ITE estimation, the Counterfactual Regression (CFR) method proposed by Shalit et al. [3] uses a fully connected network (FCN) $\phi \left(x_{i}\right)$ to map $x_{i}$ into a representation space first. Then, taking the idea of separating the learning for T=1 and T=0 as mentioned above, the CFR method uses two FCNs to predict ${\hat{y}}_{i}^{t_{i}=1}$ and ${\hat{y}}_{i}^{t_{i}=0}\;$based on $D_{T=1}$ and $D_{T=0}$, respectively, and finally optimizes the three FCNs by minimizing a CFR loss function defined as the weighted summation of $\sum^{\;}{\left[y_{i}^{t_{i}=1}-{\hat{y}}_{i}^{t_{i}=1}\right]}^{2}$ for $x_{i}\in D_{T=1}$, $\sum^{\;}{\left[y_{i}^{t_{i}=0}-{\hat{y}}_{i}^{t_{i}=0}\right]}^{2}$ for $x_{i}\in D_{T=0}$, and $IPM\left(p_{\phi }^{T=1},p_{\phi }^{T=0}\right)$, where the first two items obviously measure the estimation errors for the data from $D_{T=1}$ and $D_{T=0}$, respectively, and $IPM\left(p_{\phi }^{T=1},p_{\phi }^{T=0}\right)$ represents the integral probability metric (IPM) between the probability distributions of $D_{T=1}$ and $D_{T=0}$ in the representation space, whose minimization means balancing the impact of the confounders on the two distributions in the representation space. As mentioned in [1], the CFR model is also extended to some other improved models, such as those in [6,7]. Additionally, considering the unmeasured confounders, Anpeng Wu et al. propose an instrumental variable-based counterfactual regression method, which can also be regarded as an improvement to the CFR model [5].

ITE estimation methods based on representation learning (including the CFR method mentioned above) face the censoring problem when they are applied to survival time, because the output $y_{i}^{t_{i}=1}$ or $y_{i}^{t_{i}=0}$ denoting survival time in this case will become unavailable (also referred to as censored) if the follow-up of the patient i has been lost or patient i is still alive before the trial ends [4,8,9]. To make the methods applicable to survival data, Chapfuwa et al. proposed a Counterfactual Survival Analysis (CSA) method [4] which improves CFR by replacing the censored outputs (i.e., the survival times) with the so-called observed censoring times (please refer to the notations in Section 2.1) and revising the corresponding estimation error items in the loss function.

Although balancing the confounders helps to reduce selection bias, the above-mentioned methods cannot discriminate between confounders and non-confounders (i.e., they treat all covariates in the baseline as confounders) and therefore may balance the non-confounders which do not affect the treatment assignment $t_{i}$. Shi et al. pointed out that this may lead to a decrease in prediction precision, and proposed the so-called Dragonnet to prevent balancing non-confounders [10]. Besides the three FCNs used in CFR, Dragonnet introduces an FCN to predict the treatment ${\hat{t}}_{i}\;$and further incorporates a cross-entropy distance between ${\hat{t}}_{i}$ and $t_{i}$ in the loss function, whose minimization helps to reduce the influence of the non-confounders in the representation space and therefore helps to prevent them from being balanced [10]. However, unlike CFR and CSA, the Dragonnet method has the demerits of not balancing the confounders and is not applicable to censored survival data.

Besides representation learning methods, there are also other methods for estimating treatment effects on survival time based on machine learning, such as random survival forest (RSF), COX regression, and accelerate failure time (AFT) [11,12,13], etc. In spite of their effectiveness, a common limitation of these kind of methods is they do not balance the confounders, which could lead to selection bias [4].

Actually, besides the above-mentioned methods, Randomized Controlled Trials (RCTs) [14] are always gold standards for treatment effect estimation, in which patients meeting some particular inclusion criteria are selected and randomly assigned to a treatment group and control group; then, the treatment effect is evaluated by comparing the difference between the trial results of the two groups. The RCTs will not face the problem of selection bias because the random allocation of the treatment can guarantee that the baseline distributions in the treatment group and control group are identical. Similarly, there are also Retrospective Cohort Studies (RCSs) for treatment effect estimation [15], in which the data of the treatment and control group are selected from historical data based on inclusion criteria, but RCSs still have the identical baseline distributions in the two groups to avoid selection bias. However, RCTs may not be feasible in many cases, e.g., forcing non-smokers to smoke in an RCT for smoking is against ethics [16]. Even in the cases where RCSs are feasible, the strict inclusion criteria limits the generalizability of RCTs for the patients who cannot be represented by the included ones [17,18], which is also the case for RCSs. These demerits limit the application of RCTs and RCSs.

In spite of their limitations, the conclusions obtained from accomplished RCTs or RCSs can offer valuable qualitative prior knowledge to the counterfactual learning of the ITE, because although the $ITE_{i}=\Delta_{Y_{i}}=Y_{i}^{T=1}\left(x_{i}\right)-Y_{i}^{T=0}\left(x_{i}\right)$ is counterfactual and unavailable for each patient i, it can be obtained from the results of RCTs or RCSs where, for patients with a significant treating effect, there is $\Delta_{Y_{i}}>0$ with a high probability, and for patients without a significant treating effect, there is $\Delta_{Y_{i}}=0$ (please refer to Section 4.1 for details). However, although there exists effective methods for treatment effect estimation which introduce prior knowledge, such as [19,20], which incorporates prior knowledge on the relationship between the baseline $x_{i}$ and treatment $t_{i}$, to the best of our knowledge, there is still no method for treatment effect estimation which can take advantage of prior knowledge on the ITE obtained from RCTs or RCSs.

To sum up, there are four problems which need to be considered in the estimation of the ITE on survival time, i.e., (i) how to balance the confounders to reduce selection bias; (ii) how to handle the censored survival data; (iii) how to avoid balancing the non-confounders which may lead to the decrease in prediction precision; and (iv) how to take advantage of prior knowledge on the ITE obtained from RCTs or RCSs.

Considering the situation that the existing methods have proposed solutions to problems (i)–(iii) separately, that none of them take all three problems into consideration in a single method, and that there has been no solution to problem (iv), in this paper, we first propose a new model called CSA–Dragonnet based on CSA and Dragonnet to combine CSA’s solutions to problems (i) and (ii) and Dragonnet‘s solution to problem (iii), and then propose a CSA–Dragonnet with Embedded Prior Knowledge (CDNEPK) to further incorporate the prior knowledge obtained from RCTs or RCSs.

The more important contributions of this paper come from the second part, i.e., the proposing of CDNEPK, which includes: (i) finding a way to express different kinds of prior knowledge extracted from RCTs or RCSs in a unified form; (ii) embedding prior knowledge into the CDNEPK proposed in this paper by inserting counterfactual prediction nets into CSA–Dragonnet, whose output is denoted by ${\hat{y}}_{i}^{T=1-t_{i}}$, for $t_{i}=1\;$or 0, and incorporating new loss items into the loss function, which takes advantage of prior knowledge to extract valuable bound information for ${\hat{y}}_{i}^{T=1-t_{i}}$.

The key novelty of CDNEPK compared to the existing representation learning-based ITE estimation methods lies in the counterfactual prediction introduced in CDNEPK. As explained above, to deal with the difficulty that$\;ITE_{i}≜\Delta_{Y_{i}}=Y_{i}^{T=1}-Y_{i}^{T=0}$ is counterfactual, i.e., one of $\;Y_{i}^{T=1}$ and $\;Y_{i}^{T=0}$ is counterfactual to patient i, the existing methods train ${\hat{y}}_{i}^{t_{i}=1}=f_{T=1}\left(x_{i}^{t_{i}=1}\right)$ based on $D_{T=1}$ (i.e., that dataset of patients who have actually received treatment T=1) and ${\hat{y}}_{i}^{t_{i}=0}=f_{T=0}\left(x_{i}^{t_{i}=0}\right)$ based on $D_{T=0}$ (i.e., that dataset of patients who have actually received treatment T=0) separately, both of which have the ground truth outputs (i.e., $y_{i}^{t_{i}=1}$ and $y_{i}^{t_{i}=0}$, or their corresponding observed censoring times). While in CDNEPK, besides them, ${\hat{y}}_{i}^{T=1-t_{i}}=f_{T=1-t_{i}}\left(x_{i}^{t_{i}=*}\right)$ for * = 1 and 0, i.e., ${\hat{y}}_{i}^{T=0}=f_{T=0}\left(x_{i}^{t_{i}=1}\right)$ and ${\hat{y}}_{i}^{T=1}=f_{T=1}\left(x_{i}^{t_{i}=0}\right)$ are further introduced and trained, which are called the counterfactual prediction because $T=1-t_{i}$ has not actually happened and there are no ground truth data for ${\hat{y}}_{i}^{T=0}$ or ${\hat{y}}_{i}^{T=1}$. However, as will be explained in Section 4.3, we can extract valuable bound information for the prediction of ${\hat{y}}_{i}^{T=0}$ or ${\hat{y}}_{i}^{T=1}$ from prior knowledge yielded by RCTs and RCSs, so we add the counterfactual prediction nets for ${\hat{y}}_{i}^{T=0}=f_{T=0}\left(x_{i}^{t_{i}=1}\right)$ and ${\hat{y}}_{i}^{T=1}=f_{T=1}\left(x_{i}^{t_{i}=0}\right)$ and their corresponding loss items for training to take full advantage of the valuable information offered by prior knowledge.

This paper is organized as follows. Section 2 first defines the notations and gives a brief introduction to CSA and Dragonnet. Then, based on CSA and Dragonnet, CSA–Dragonnet is proposed in Section 3 to handle the problems (i)–(iii) mentioned above simultaneously. In Section 4, we formulate a unified expression of the prior knowledge yielded by RCTs and RCSs and propose CDNEPK with incorporated counterfactual prediction branches and its corresponding loss items. Semi-synthetic data experiments are designed to test the performance of the proposed methods in Section 5. Real-world experiments based on Hepatocellular Carcinoma data covering 1459 patients in China are used to show the potential usage of CDNEPK. Finally, we draw a conclusion in Section 7.

## 2. Notations and Preliminary

### 2.1. Notations and Description of Dataset

Throughout the paper, we use the following notations:

1. Let T=1 or 0 denote two treatments for comparison. For patient i, let $Y_{i}^{T=1}$ and $Y_{i}^{T=0}$ represent the potential outcomes of treatment T=1 and T=0, respectively; let $y_{i}$, $x_{i}$, and $t_{i}$ denote the observed survival time, baseline vector comprising m covariates, and the actual treatment patient i has received, respectively; let $x_{i}^{t_{i}=*}$ and $y_{i}^{t_{i}=*}\;$denote that $y_{i}\;$and $x_{i}$ are corresponding to an actual treatment $t_{i}=\;*$ (i.e., the observed survival time and the baseline of patient i who has received a treatment $t_{i}=*$, ∗ =1 or 0). For the case where we do not need to refer to the specific value of $t_{i}$, we also use $x_{i}^{t_{i}}$ and $y_{i}^{t_{i}}$ for short.
2. Considering the censoring problem, let $y_{cen,i}$ denote the observed censoring time when $y_{i}$ is censored, which is defined as “the time up to which we are certain that the event has not occurred” according to [4], where the event refers to death here. To denote $y_{i}$ and $y_{cen,i}$ in a unified way, like reference [9], let $\gamma_{i}$ denote the observed time, which equals $y_{i}\;$when it is available, and is set at $y_{cen,i}$ when $y_{i}$ is censored. Similar to the meaning of $y_{i}^{t_{i}=*}$, we use $\gamma_{i}^{t_{i}=*}$ to denote the observed time of patient i who has received an actual treatment $t_{i}=*$. Let $\delta_{i}=0\left(1\right)\;$indicate that survival time is (is not) censored.
3. Let $D_{all}=\left\{\left(\gamma_{i},x_{i},\delta_{i},t_{i}\right),i=1,2,...,N\right\}$ denote the historical dataset of all patients and let $D_{T=*}=\left\{\left(\;\gamma_{i}^{t_{i}=*},\;x_{i}^{t_{i}=*},\delta_{i}\right),i=1,...,N_{T=*}\right\}$ represent the historical dataset for patients who have received treatments T=∗, with ∗=1 or 0, where $D_{T=1}$ and $D_{T=0}$ are the subsets of $D_{all}$, with $N=N_{T=1}+N_{T=0}$.
4. Let ${\hat{t}}_{i}$ denote the prediction of $t_{i}$ based on $x_{i}$ and ${\hat{y}}_{i}^{t_{i}=*}$ denote the prediction of survival time$\;y_{i}^{t_{i}=*}$ based on $x_{i}^{t_{i}=*}$, with ∗=1 or 0, which is also called factual prediction. While on the contrary, we use ${\hat{y}}_{i}^{T=1-t_{i}}$ to represent a counterfactual prediction, which uses $x_{i}^{t_{i}=*}$ (for a patient who has received $t_{i}=*$) to predict what will happen if the contrary treatment $T=1-t_{i}$ is adopted (please see Section 4.2 for details).
5. Let $Y_{i}^{T=*}$ represent the potential outcome of T=∗ (with ∗=1 or 0) and call $ITE_{i}≜\Delta_{Y_{i}}=Y_{i}^{T=1}-Y_{i}^{T=0}$ the Individual Treatment Effect of T=1 relative to T=0 [1]. In this paper, suppose we have historical datasets $D_{all}$, $D_{T=1}$, and $D_{T=0}$, and some prior knowledge which can be expressed by $\Delta_{Y_{i}}>0$ for $x_{i}\in \Omega$ and $\Delta_{Y_{i}}=0$ for $x_{i}\in \Gamma$ (please refer to Section 4.1 for details).

### 2.2. A Brief Introduction to CSA and Dragonnet

CSA [4] contains seven FCNs, i.e., ϕ, $h_{T=1}$, $h_{T=0}$, $u_{T=1}$, $u_{T=0}$, $g_{T=1}$, and $g_{T=0}$. Among them, $\phi \left(x_{i}\right)$ with $x_{i}$ as the input is first used to map $x_{i}$ into a representation space, then $\phi \left(x_{i}^{t_{i}=1}\right)\;$and $\phi \left(x_{i}^{t_{i}=0}\right)$ are further fed into two branches to predict ${\hat{y}}_{i}^{t_{i}=1}$ and ${\hat{y}}_{i}^{t_{i}=0}$, respectively, with ${\hat{y}}_{i}^{t_{i}=1}=h_{T=1}\left(g_{T=1}\left(\phi \left(x_{i}^{t_{i}=1}\right)\right)\;⨁\;u_{T=1}\left(\varepsilon_{1}\right)\right)$ for patients from $D_{T=1}$ and ${\hat{y}}_{i}^{t_{i}=0}=h_{T=0}\left(g_{T=0}\left(\phi \left(x_{i}^{t_{i}=0}\right)\right)⨁\;u_{T=0}\left(\varepsilon_{0}\right)\right)$ for patients from $D_{T=0}$, where Δ denotes the concatenating operation and $\varepsilon_{1}$ and $\varepsilon_{0}$ are random input vectors. The following CSA loss is minimized in [4] to train the seven FCNs:

(1) $$\;L_{CSA}=\sum_{j=0,1}^{}\sum_{x_{i}\in D_{T=j}}\frac{1}{N_{T=j}}\left[\delta_{i}\left|\gamma_{i}^{t_{i}=j}-{\hat{y}}_{i}^{t_{i}=j}\right|+(1-\delta_{i})\max\left(0,\gamma_{i}^{t_{i}=j}-{\hat{y}}_{i}^{\;t_{i}=j}\right)\right]+\alpha IPM(p_{\phi }^{T=1},p_{\phi }^{T=0})$$

where $\delta_{i}\left|\;\gamma_{i}^{t_{i}=j}-{\hat{y}}_{i}^{t_{i}=j}\right|$ and $\max\left(0,\;\gamma_{i}^{t_{i}=j}-{\hat{y}}_{i}^{t_{i}=j}\right)$ are used to measure the error between the estimated output ${\hat{y}}_{i}^{t_{i}}$ and the observed time$\;\gamma_{i}^{t_{i}}$, which may become more controversial if the survival data is censored, and $IPM\left(p_{\phi }^{T=1},p_{\phi }^{T=0}\right)$ represents the distance between the distributions of $\phi \left(x_{i}^{t_{i}=1}\right)$ and $\phi \left(x_{i}^{t_{i}=0}\right)$, which actually reflects the impact of selection bias in the representation space caused by the confounders.

Dragonnet [10] consists of four FCNs, i.e., ϕ, $h_{T=1}$, $h_{T=0}$, and ψ. Similar to the CSA model, $\phi \left(x_{i}\right)$ is still used to map $x_{i}$ into a representation space; ${\hat{y}}_{i}^{t_{i}=1}=h_{T=1}\left(\phi \left(x_{i}^{t_{i}=1}\right)\right)$ and ${\hat{y}}_{i}^{t_{i}=0}=h_{T=0}\left(\phi \left(x_{i}^{t_{i}=0}\right)\right)$ are used to predict the outcomes for patients from $D_{T=1}$ and $D_{T=0}$, respectively. Unlike the CSA model, a new FCN ψ is introduced in Dragonnet to predict the treatment ${\hat{t}}_{i}$ by ${\hat{t}}_{i}=\psi \left(\phi \left(x_{i}\right)\right)$, and the loss to be minimized is defined by:

(2) $$L_{\mathrm{Dragonnet}}=\frac{1}{N_{T=1}}\sum_{x_{i}\in D_{T=1}}{\left[y_{i}^{t_{i}=1}-{\hat{y}}_{i}^{t_{i}=1}\right]}^{2}+\frac{1}{N_{T=0}}\sum_{x_{i}\in D_{T=0}}{\left[y_{i}^{t_{i}=0}-{\hat{y}}_{i}^{t_{i}=0}\right]}^{2}+\beta \sum_{x_{i}\in D_{all}}CE\left({\hat{t}}_{i},t_{i}\right)$$

where the first two items are estimation errors of the outcomes, $\underset{i\in D_{all}}{\sum }CE\left({\hat{t}}_{i},t_{i}\right)$ is the average cross-entropy distance between ${\hat{t}}_{i}$ and $t_{i}$ over all patients which reflects the impact of the non-confounders on ${\hat{t}}_{i}$. So, its minimization helps to reduce the influence of the non-confounders in the representation space and prevents them from being balanced.

From (1) and (2) it can be seen that for the four problems needed to be considered in the estimation of ITE on survival time mentioned in Section 1, the CSA method gives solutions to problem (i) and (ii), i.e., how to balance the confounders to reduce selection bias and how to handle the censored survival times data, and the Dragonnet method gives a solution to problem (iii), i.e., how to avoid balancing the non-confounders which may lead to the decrease in prediction precision.

## 3. CSA–Dragonnet

As summarized in Section 1, for the ITE estimation, the CFR model [3] is proposed to reduce selection bias by balancing the confounders in the representation space, and the CSA method [4] is proposed by extending CFR to handle the survival data which could be censored. Since CFR does not discriminate between the confounders and the non-confounders, it also balances the non-confounders and leads to a decrease in prediction precision. Hence, Dragonnet is proposed in [10] to reduce the influence of the non-confounders in the representation space and to prevent them from being balanced. However, Dragonnet still suffers from the problems of selection bias and censoring [10]. So in this section, we propose a CSA–Dragonnet based on the CSA model [4] and Dragonnet [10] to combine their advantages.

**Figure 1:**
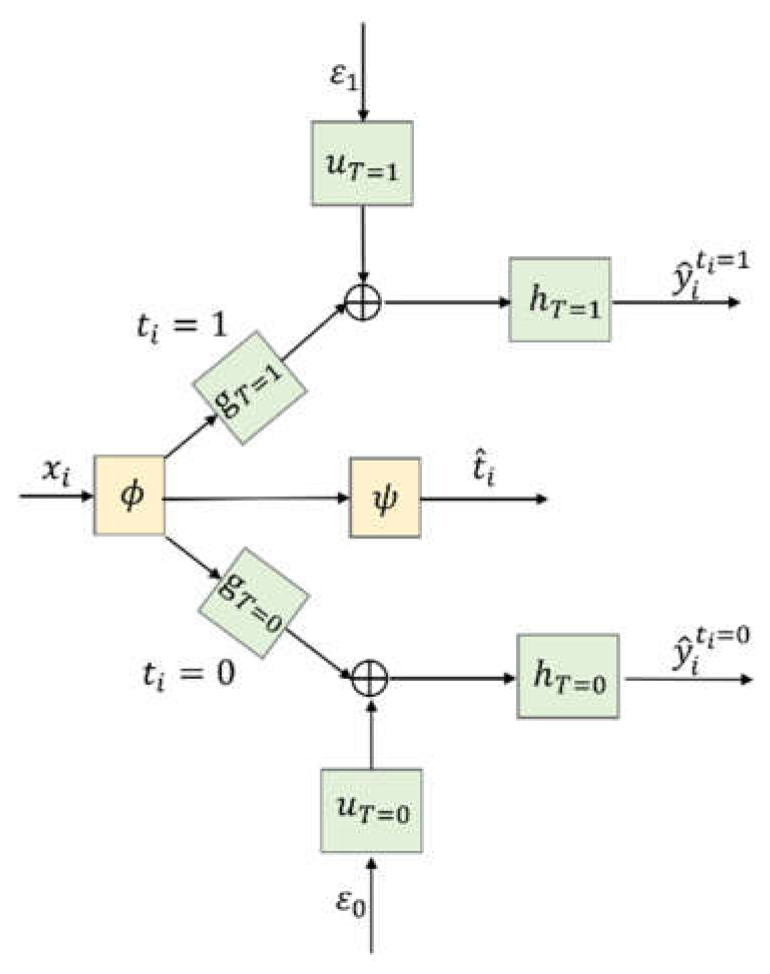
CSA–Dragonnet.

Figure 1 shows the architecture of the proposed CSA–Dragonnet. The CSA–Dragonnet consists of three parts, i.e., (i) as in the CSA [4] and Dragonnet [10] models, the baseline $x_{i}$ of all patients from $D_{all}$ is mapped onto a latent representation by an FCN $\phi \left(x_{i}\right);$ (ii) as in Dragonnet [10], in order to reduce the influence of the non-confounders in the representation space, a single-layered FCN ψ with $\phi \left(x_{i}\right)$ as the input is used to predict the probability of the treatment, i.e.,$\;{\hat{t}}_{i}=\psi \left(\phi \left(x_{i}\right)\right)$; (iii) as in the CSA model [4], in order to predict ${\hat{y}}_{i}^{t_{i}=1}$ and ${\hat{y}}_{i}^{t_{i}=0}$, $\phi \left(x_{i}\right)$ of all the patients are divided into two parts, i.e., $\phi \left(x_{i}^{t_{i}=1}\right)\;$for patients from $D_{T=1}$ and $\phi \left(x_{i}^{t_{i}=0}\right)$ for patients from $D_{T=0}$, which are further fed into two groups of networks on the top and bottom branches of Figure 1, respectively. In the two branches, $g_{T=*}$, $h_{T=*}$, and $u_{T=*}$ (with * = 1 or 0) are all FCNs, $\varepsilon_{1}$ and $\varepsilon_{0}$ are specially designed random inputs [4], and ⨁ denotes the concatenating operation rather than summation, i.e., the input of $h_{T=*}$ is a vector composed of $g_{T=*}\left(\phi \left(x_{i}\right)\right)$ and $u_{T=*}\left(\varepsilon_{*}\right)$. The random inputs $\varepsilon_{1}$ and $\varepsilon_{0}$ are utilized to introduce some randomness model in the time generation process [4]. Please refer to reference [4] for the details of the non-parametric survival model.

In summary, the whole CSA–Dragonnet is a three-head neural network, in which the inputs include the baseline and two random sources, and the outputs include the predicted probability of the treatment ${\hat{t}}_{i}$ as well as the predicted survival times ${\hat{y}}_{i}^{t_{i}=1}$ and ${\hat{y}}_{i}^{t_{i}=0}$. Eight FCNs are involved in the network, among which ϕ and ψ are shared networks for all patients, while $u_{T=1}$,$\;g_{T=1}$,$\;h_{T=1}$ on the top branch are only applicable to patients with $t_{i}=1$ and $u_{T=0}$,$\;g_{T=0}$,$\;h_{T=0}$ on the bottom branch are only applicable to patients with $t_{i}=0$. FCNs ϕ,$\;g_{T=1}$,$g_{T=0}$ are defined by Leaky Rectified Linear Unit (Relu) activation functions; FCNs $u_{T=1}$,$u_{T=0}$ use Hyperbolic Tangent(tanh) activation functions; FCNs $h_{T=1}$,$\;h_{T=0}$ are defined by exponential activation functions; and ψ is defined by the softmax activation function.

The loss function to train the eight FCNs in CSA–Dragonnet can be defined by combining (1) for CSA and (2) for Dragonnet as follows:

(3) $$\begin{array}{l} L_{CSA-Dragon}=\sum_{j=0,1}^{}\sum_{x_{i}\in D_{T=j}}\frac{1}{N_{T=j}}\left[\delta_{i}\left|\gamma_{i}^{t_{i}=j}-{\hat{y}}_{i}^{t_{i}=j}\right|+(1-\delta_{i})\max\left(0,\gamma_{i}^{t_{i}=j}-{\hat{y}}_{i}^{\;t_{i}=j}\right)\right]\\ \;\;+\alpha IPM(p_{\phi }^{T=1},p_{\phi }^{T=0})+\beta \sum_{i\in D_{all}}CE\left({\hat{t}}_{i},t_{i}\right) \end{array}$$

where the last item comes from (2) (i.e., the loss function of Dragonnet) and the other items come from (1) (i.e., the loss function of the CSA method). Please refer to references [21] and [22] for detailed definitions of the IPM distance and cross-entropy distance, respectively.

As explained in Section 1, (i) $IPM\left(p_{\phi }^{T=1},p_{\phi }^{T=0}\right)$ measures the difference between the impacts of the confounders in the representation space, so minimizing it helps to balance the confounders and reduces selection bias [3,4]; (ii) $\underset{i\in D_{all}}{\sum }CE\left({\hat{t}}_{i},t_{i}\right)$ measures the impact of the non-confounders on ${\hat{t}}_{i}$, so its minimization helps to reduce the influence of the non-confounders in the representation space and prevents them from being balanced [10]; (iii) as for the first part, since there is $\delta_{i}=1$ and $\gamma_{i}^{t=j}=y_{i}^{t=j}$ for patients with observed survival time, minimizing the summation of $\delta_{i}\left|\gamma_{i}^{t=j}-{\hat{y}}_{i}^{t=j}\right|$ means encouraging ${\hat{y}}_{i}^{t=j}$ to be close to the ground truth, while since $\delta_{i}=0$ and $\gamma_{i}^{t=j}$ is set as the observed censoring time $y_{cen,i}$ for patients whose survival times are censored, minimizing the summation of $\left(1-\delta_{i}\right)\max\left(0,\gamma_{i}^{t=j}-{\hat{y}}_{i}^{t=j}\right)$ means encouraging ${\hat{y}}_{i}^{t=j}\;\left(j=1,0\right)$ to exceed the observed censoring time [4]. Hence, CSA–Dragonnet with the loss $L_{CSA-Dragon}$ can balance the confounders, handle the censored survival data, and avoid balancing the non-confounders simultaneously.

**Remark 1.**  *CSA–Dragonnet is a combination of CSA and Dragonnet. (i) It will reduce to the CSA model* [4] *if the middle branch for* ${\hat{t}}_{i}$ *in* Figure 1 *and the cross-entropy distance* $\underset{i\in D_{all}}{\sum }CE\left({\hat{t}}_{i},t_{i}\right)$ *in (3) are removed; (ii) CSA–Dragonnet will reduce to Dragonnet if* ϕ *is directly connected to* $h_{T=1}$ *and* $h_{T=0}$ *in the top and bottom branches (i.e.,*$\;u_{T=1}$*,*$\;g_{T=1}$*,*$\;u_{T=0}$*,*$\;g_{T=0}$*,* $\varepsilon_{1}$ *and* $\varepsilon_{0}$ *are removed), the IPM distance between the distributions of* $\phi \left(x_{i}^{t_{i}=1}\right)$ *and* $\phi \left(x_{i}^{t_{i}=0}\right)$ *is removed, and the first part of* $L_{CSA-Dragon}$ *is replaced with* $\underset{j=0,1}{\sum }\underset{i\in D_{T=j}}{\sum }{\left(y_{i}^{t=j}-{\hat{y}}_{i}^{t=j}\right)}^{2}$*, which cannot handle censored data*.

## 4. CSA–Dragonnet with Embedded Prior Knowledge (CDNEPK)

### 4.1. A Unified Expression of the Prior Knowledge Yielded by RCTs and RCSs

As mentioned in Section 1, the results of RCTs or RCSs can offer valuable information about the ITE to counterfactual learning. In the following, two examples are given to illustrate it in detail.

**Example** **1.** *McNamara et al. investigated if advanced Hepatocellular Carcinoma (HCC) patients with liver function in good condition could benefit from “sorafenib” [23] through a systematic review and meta-analysis of 30 related studies based on RCTs or RCSs, which comprised 8678 patients altogether. The conclusion was that patients with Child-Pugh grade A could benefit from “sorafenib” significantly, while the effect of “sorafenib” on patients with Child-Pugh grade B is still controversial [23].*

The Child-Pugh (CP) grades mentioned in the conclusion are widely used to describe the liver functional status of a patient [23]. It is determined by a CP score defined as the summation of the scores of five covariates in the baseline listed in Table 1, i.e., the covariate scores of hepatic encephalopathy (HE), ascites (AC), total bilirubin (TBIL), albumin (ALB), and prothrombin time (PT), which are further assigned according to the conditions given in Table 1 [24,25]. More concretely, each row of Table 1 gives the rules for how to assign a score to a corresponding covariate listed in the first column. In addition, a CP score of five or six is also banded into the CP grade A, and a CP score of seven, eight, or nine is banded into the CP grade B [24,25].

**Example** **2.**  *Wang et al. investigated whether patients with small HCC could benefit from a hepatectomy through a retrospective control study [26]. A total of 143 patients with HCC were involved in the trial, all of whom satisfied the inclusion criterion of “with single tumor lower than 2 cm, no distant metastasis (DM), no vascular invasion (VI), and no ascites (AC)”. Comparisons between the results of the hepatectomy and control groups showed that the hepatectomy could not significantly extend survival time for patients satisfying the inclusion criterion.*

Let $Y_{i}^{T=1\left(0\right)}$ denote the potential survival time of patient i receiving “sorafenib” (not receiving “sorafenib”), the conclusions in Example 1 actually tell us that if a patient *i* belongs to the CP grade A, then there is $ITE_{i}=\Delta_{Y_{i}}=Y_{i}^{T=1}-Y_{i}^{T=0}>0$ with a high certainty even if the patient was not involved in the meta-analysis conducted by [23]. This prior knowledge offers valuable information on the counterfactual ITE to the patients involved in a representation learning. Similarly, let $Y_{i}^{T=1\left(0\right)}$ represent the potential survival time of patient i receiving a hepatectomy (not receiving a hepatectomy), we know from Example 2 that if patient i meets the condition of “with single tumor lower than 2 cm, no distant metastasis (DM), no vascular invasion (VI), and no ascites”, there is $\Delta_{Y_{i}}=0$ with a high possibility, which is also important prior knowledge for representation learning.

It can be seen that Examples 1 and 2 describe the conditions of the knowledge in different ways. In Example 2, the original covariates in the baseline are directly evaluated in the inclusion criterion, which is common in RCTs or RCSs, while in Example 1, the CP score derived from the original covariates in the baseline is evaluated in the condition. This is also a way of representativeness to express the conditions of the patients, because besides the CP score adopted in Example 1, there are also many other different kinds of scores to measure the initial conditions of the patients related to various diseases, which may influence the further treatment effects, such as the influence of the lung allocation score [27] on lung transplantation [28] and the influence of the renal score [29] on renal cryoablation [30], etc.

In the following, we define a set to denote the group of patients satisfying the two typical kinds of conditions mentioned above in a unified way, i.e.,

(4) $$\Theta =\left\{x_{i}|\sum_{j=1}^{m}\sum_{l=1}^{d}{ο}_{}^{j,l}I(\lambda_{}^{j,l}\leq x_{i,j}^{}\leq \mu_{}^{j,l})\in V\right\}$$

where $x_{i,j}$ denotes the jth element of $x_{i}$ and I(*) is an indicative function which equals 1(0) when the inequality in the brackets holds (does not hold). The number of indicative functions (i.e., d), the weighting coefficients $o^{j,l}$ for j=1…m and l=1,…,d*,* the thresholds $\lambda^{j,l}$, $\mu^{j,l}$ for j=1,…,m and l=1,…,d, and the set V should be determined by the specific conditions of the corresponding knowledge.

Formula (4) can cover both of the two examples given previously. If we let d=3, $o^{j,1}=1$, $o^{j,2}=2$, $o^{j,3}=3$, it is obvious that the group of patients with the CP grade A mentioned in Example 1 can be described by:

(5) $$\Theta_{\Delta >0}=\left\{x_{i}|\sum_{j\in \left\{\mathrm{HE},\mathrm{AC},\mathrm{TBIL},\mathrm{ALB},\mathrm{PT}\right\}}\left[I\left(\lambda^{j,1}\leq x_{i,j}\leq \mu^{j,1}\right)+2I\left(\lambda^{j,2}\leq x_{i,j}\leq \mu^{j,2}\right)+3I\left(\lambda^{j,3}\leq x_{i,j}\leq \mu^{j,3}\right)\right]\in \left\{5,6\right\}\right\}$$

where the coefficients $\lambda^{j,l}$ and $\mu^{j,l}$ for j∈{HE, AC, TBIL,ALB,PT} and *l* = 1, 2, and 3 can be assigned according to Table 1. For example, it is direct that there are $\lambda^{HE,2}=1$, $\mu^{HE,2}=2$; $\lambda^{AC,2}=\mu^{AC,2}=1$;$\;\lambda^{PT,1}=0,$ $\mu^{PT,1}\;$=4, and $\lambda^{TBIL,3}=51,$ $\mu^{TBIL,3}=+\infty$. As for Example 2, the group of patients satisfying the inclusion criterion of “with single tumor lower than 2 cm, no distant metastasis (DM), no vascular invasion (VI), and no ascites (AC)” can be directly written as:

(6) $$\Theta_{\Delta =0}=\left\{x_{i}|I(x_{i,\mathrm{diameter}}\leq 2)+I(x_{i,\mathrm{number}}=1)+I(x_{i,DM}=0)+I(x_{i,VI}=0)+I(x_{i,AC}=0)\in \left\{5\right\}\right\}$$

where $x_{i,j}$ denotes the jth element of $x_{i}$ for j∈{diameter, number, DM,VI,AC}.

So, the knowledge obtained in Examples 1 and 2 can be written as $ITE=\Delta_{Y_{i}}>0$, if $x_{i}\in \Theta_{\Delta >0}$ and $ITE=\Delta_{Y_{i}}=0$, if $x_{i}\in \Theta_{\Delta =0}$. Now consider a general situation: suppose we can obtain $ITE=\Delta_{Y_{i}}>0$, if $x_{i}\in \Theta_{\Delta >0}^{\tau }$ for τ=1,…,s, and $ITE=\Delta_{Y_{i}}=0$, if $x_{i}\in \Theta_{\Delta >0}^{\rho }$ for ρ=1,…,ϱ, then let:

(7) $$\Omega =\cup_{\tau =1}^{s}\Theta_{\Delta >0}^{\tau }\;\;\;\;\Gamma =\cup_{\rho =1}^{\varrho }\Theta_{\Delta =0}^{j}$$

where $\cup^{\;}$ represents the union. The prior knowledge can be finally written as

(8) $$\left\{ \begin{array}{l} \Delta_{Y_{i}}>0,{\;\mathrm{if}\;x}_{i}\in \Omega \;\\ \Delta_{Y_{i}}=0,{\;\mathrm{if}\;x}_{i}\in \Gamma \; \end{array}\right.$$

### 4.2. Importance of Counterfactual Prediction

As shown in Figure 1, during the training process, when predicting ${\hat{y}}_{i}^{t_{i}}$ for patient i who has really received treatment $t_{i}$, CSA–Dragonnet feeds the representation $\phi \left(x_{i}^{t_{i}}\right)$ into either the top branch or the bottom branch according to the actual value of $t_{i}$, i.e.,$\;\phi \left(x_{i}^{t_{i}}\right)$ is only fed into the branch consisting of $g_{T=t_{i}}$, $h_{T=t_{i}}$, and $u_{T=t_{i}}$ because the observed time $\gamma_{i}^{t_{i}}$ is available for that branch and can serve as the ground truth label (when $y_{i}^{t_{i}}$ is not censored) or at least as a bound for ${\hat{y}}_{i}^{t_{i}}$ (when the true survival time $y_{i}^{t_{i}}$ is censored) according to the loss (3).

As defined in Notation (4) in Section 2.1, if $\phi \left(x_{i}^{t_{i}}\right)\;$is fed into another branch composed of $g_{T=1-t_{i}}$, $h_{T=1-t_{i}}$, and $u_{T=1-t_{i}}$, the output is denoted by ${\hat{y}}_{i}^{T=1-t_{i}}$ and is called the counterfactual prediction because the treatment $1-t_{i}$ has not happened to patient i. The reason why ${\hat{y}}_{i}^{T=1-t_{i}}$ is not calculated in the existing representation methods is that $\gamma_{i}^{T=1-t_{i}}$ is counterfactual and therefore there is no ground truth information for training the model in that situation.

Now let us discuss what benefit the prior knowledge from formula (8) will bring to the ITE estimation. Although for each patient i in the historical dataset, of the two potential outputs $Y_{i}^{T=1}$ and $Y_{i}^{T=0}$, one must have the corresponding observed time $\gamma_{i}^{t_{i}}$ and the other one must be counterfactual, so the knowledge $\Delta_{Y_{i}}=Y_{i}^{T=1}-Y_{i}^{T=0}>0$ or $\Delta_{Y_{i}}=Y_{i}^{T=1}-Y_{i}^{T=0}=0$ obviously may offer additional information on the counterfactual potential output, which can be further used as some kind of bound for the counterfactual prediction ${\hat{y}}_{i}^{T=1-t_{i}}$.

So, in order to take full advantage of the prior knowledge given by RCTs or RCSs, in the following, we will first enhance the CSA–Dragonnet by incorporating counterfactual prediction branches which can output ${\hat{y}}_{i}^{T=1-t_{i}}$ and further by introducing new items into the loss function to guide the training of the counterfactual prediction outputs. We refer to the enhanced method as CSA–Dragonnet with Embedded Prior Knowledge (CDNEPK).

### 4.3. Architecture of CDNEPK with Incorporated Counterfactual Prediction Branches

**Figure 2:**
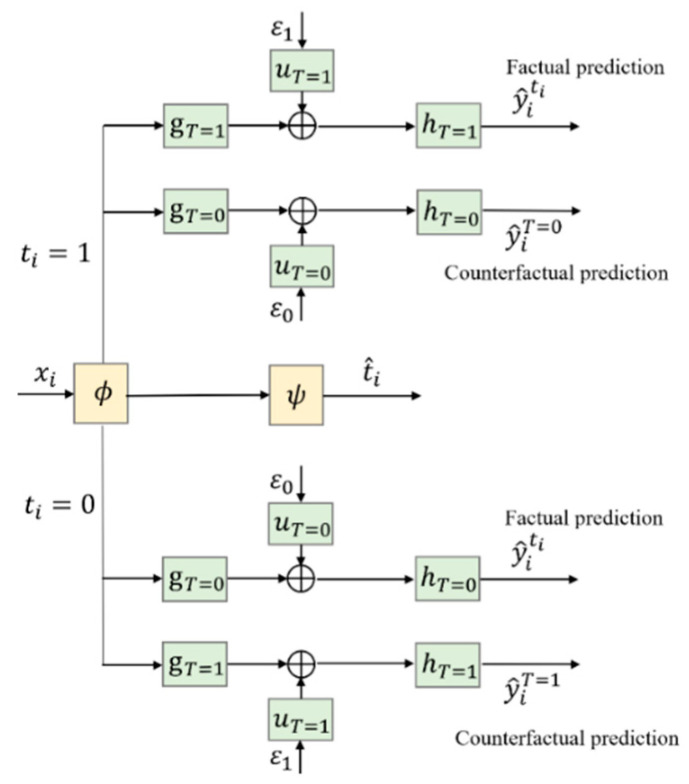
Introducing counterfactual prediction branches into CSA–Dragonnet.

To support the counterfactual prediction, two new branches to predict ${\hat{y}}_{i}^{T=1-t_{i}}$ for $t_{i}=1$ and 0, i.e., ${\hat{y}}_{i}^{T=0}$and ${\hat{y}}_{i}^{T=1}$, can be added to CSA–Dragonnet, as shown in Figure 2.

**Figure 3:**
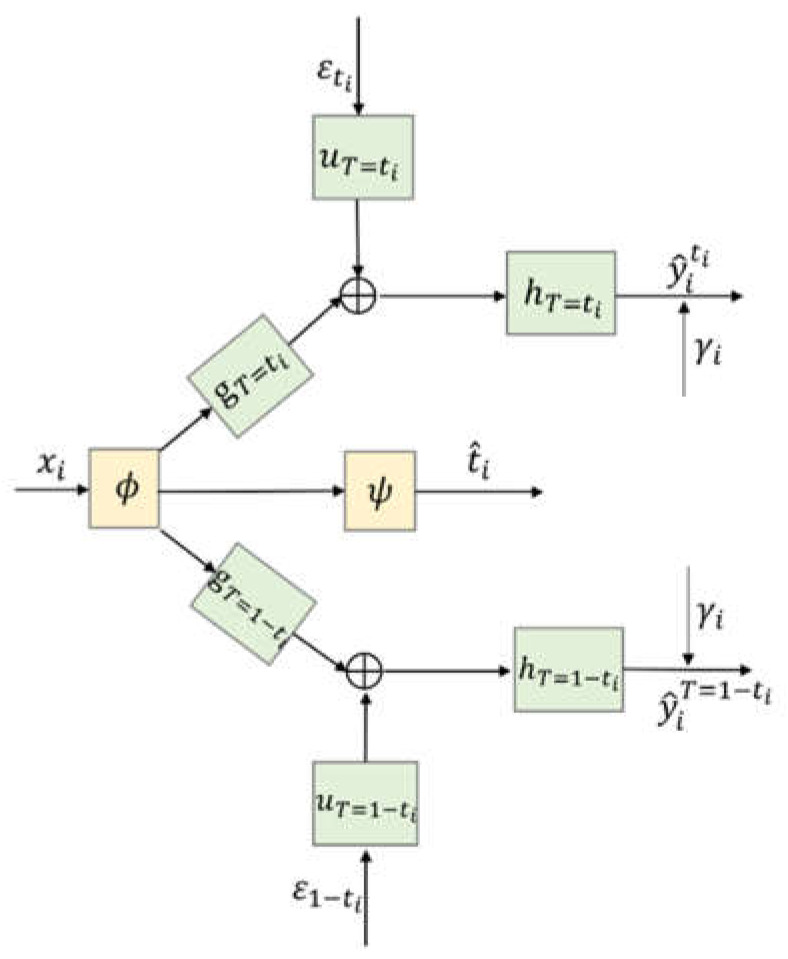
Architecture of CDNEPK.

Figure 3 gives a more concise diagram for the CDNEPK, which is equivalent to Figure 2. In Figure 3, the top branch is for factual prediction, which actually combines the calculations in both the top and bottom branches of Figure 1 (or the top and 4th branches of Figure 2) into one branch. Similarly, the bottom branch of Figure 3 is for counterfactual prediction, which combines the 2nd and 5th branches of Figure 2. For convenience, for a patient i who has received treatment $t_{i}$, we call the top branch of Figure 3 (which consists of $g_{T=t_{i}}$, $h_{T=t_{i}}$, and $u_{T=t_{i}})$ the factual prediction branch, and call the bottom branch of Figure 3 (which consists of $g_{T=1-t_{i}}$, $h_{T=1-t_{i}}$, and $u_{T=1-t_{i}})$ the counterfactual prediction branch hereafter.

### 4.4. Loss Items of CDNEPK with Incorporated Prior Knowledge

As explained in Section 4.2, the prior knowledge $\Delta_{Y_{i}}$ $=Y_{i}^{T=1}-Y_{i}^{T=0}>0$ for $x_{i}\in \Omega$ and $\Delta_{Y_{i}}$ $=Y_{i}^{T=1}-Y_{i}^{T=0}=0\;$for $x_{i}\in \Gamma$ offers valuable information for the training of the bottom counterfactual prediction branch. In this section, we will discuss how to incorporate this information into the loss function according to different situations of $t_{i}=1$ or 0 (i.e., whether patient i has actually received the treatment or not), $\delta_{i}=0$ or 1 (i.e., whether the patient’s survival time is censored or not), and $\Delta_{Y_{i}}>0\;$or $\Delta_{Y_{i}}=0$ (i.e., whether patient i could greatly benefit from the treatment T=1 relative to T=0 or not according to prior knowledge).

1. Patients with prior knowledge $\Delta_{Y_{i}}>0$ ($x_{i}\in \Omega$)
  (i) $t_{i}=0$, $\delta_{i}=1$ and $\Delta_{Y_{i}}>0$.

In this case, since the survival time is not censored, we know that of the two potential outputs $Y_{i}^{T=1}$ and $Y_{i}^{T=0}$ in the prior knowledge, $Y_{i}^{T=0}$ has the ground truth observation, i.e., there is $Y_{i}^{T=0}=\gamma_{i}^{t_{i}=0}$ and $\gamma_{i}^{t_{i}=0}$ is the true survival time, but $Y_{i}^{T=1}$ is counterfactual. Then, the prior knowledge $Y_{i}^{T=1}-Y_{i}^{T=0}>$0 for $x_{i}\in \Omega$ is equivalent to $Y_{i}^{T=1}>Y_{i}^{T=0}=\gamma_{i}^{t_{i}=0}$, which means $\gamma_{i}^{t_{i}=0}$ can be used as a lower bound of the counterfactual prediction of ${\hat{y}}_{i}^{T=1-t_{i}}={\hat{y}}_{i}^{T=1}$, or in other words, there should be a constraint ${\hat{y}}_{i}^{T=1-t_{i}}={\hat{y}}_{i}^{T=1}>\gamma_{i}^{t_{i}=0}$ for predicting ${\hat{y}}_{i}^{T=1}$. Let $N_{x_{i}\in \Omega }$ denote the number of patients who belong to Ω, we can define the following loss item:

(9) $$L_{11}=\frac{N_{x_{i}\in \Omega }}{N}\sum_{x_{i}\in D_{T=0}\cap \Omega }\delta_{i}\max(0,\gamma_{i}^{t_{i}=0}-{\hat{y}}_{i}^{T=1-t_{i}})$$

whose minimization will penalize $\gamma_{i}^{t_{i}=0}-{\hat{y}}_{i}^{T=1-t_{i}}>0$ and favors the satisfaction of the constraint ${\hat{y}}_{i}^{T=1-t_{i}}={\hat{y}}_{i}^{T=1}>\gamma_{i}^{t_{i}=0}.$

(ii) $t_{i}=0$, $\delta_{i}=0$ and $\Delta_{Y_{i}}>0$.

In this case, the survival time is censored, which means at the end of the trial the patient is still alive. So, the observed time $\gamma_{i}^{t_{i}=0}$ must be less than the true survival time (i.e., there is $Y_{i}^{T=0}>\gamma_{i}^{t_{i}=0}$). Then, of the two potential outputs $Y_{i}^{T=1}$ and $Y_{i}^{T=0}$,$\;Y_{i}^{T=0}\;$only has a lower bound $\gamma_{i}^{t_{i}=0}$ instead of the ground truth, and $Y_{i}^{T=1}$ is still counterfactual. Since $Y_{i}^{T=0}>\gamma_{i}^{t_{i}=0}$ and $Y_{i}^{T=1}-Y_{i}^{T=0}>$ 0 lead to $Y_{i}^{T=1}>Y_{i}^{T=0}$> $\gamma_{i}^{t_{i}=0}$ for $x_{i}\in \Omega$, we still have the constraint ${\hat{y}}_{i}^{T=1-t_{i}}={\hat{y}}_{i}^{T=1}>\gamma_{i}^{t_{i}=0}$ for ${\hat{y}}_{i}^{T=1-t_{i}}\;$ and therefore can define the loss term by only replacing the $\delta_{i}$ in (9) with $\left(1-\delta_{i}\right)$ considering $\delta_{i}=0$, i.e.,

(10) $$L_{12}=\frac{N_{x_{i}\in \Omega }}{N}\sum_{x_{i}\in D_{T=0}\cap \Omega }\left(1-\delta_{i}\right)\max(0,\gamma_{i}^{t_{i}=0}-{\hat{y}}_{i}^{T=1-t_{i}})$$

It is worth mentioning that although $\gamma_{i}^{t_{i}=0}$ is used as the lower bound in both case (i) and case (ii), it is more conservative in this case than in case (i) because of $Y_{i}^{T=0}$> $\gamma_{i}^{t_{i}=0}$.

(iii) $t_{i}=1,$ $\delta_{i}=1$ and $\Delta_{Y_{i}}>0$.

In this case, $Y_{i}^{T=1}$ has the ground truth $\gamma_{i}^{t_{i}=1}$, i.e., $Y_{i}^{T=1}=\gamma_{i}^{t_{i}=1}$, with $\gamma_{i}^{t_{i}=1}\;$equaling the true survival time, but $Y_{i}^{T=0}$ is counterfactual. So, $Y_{i}^{T=1}-Y_{i}^{T=0}>0$ is equivalent to $Y_{i}^{T=1}=\gamma_{i}^{t_{i}=1}>$ $Y_{i}^{T=0}$, which means there is a constraint ${\hat{y}}_{i}^{T=1-t_{i}}={\hat{y}}_{i}^{T=0}<Y_{i}^{T=1}=\gamma_{i}^{t_{i}=1}$ for ${\hat{y}}_{i}^{T=0}$ with $\gamma_{i}^{t_{i}=1}$ as the upper bound. Then, the loss item can be defined by penalizing ${\hat{y}}_{i}^{T=1-t_{i}}-\gamma_{i}^{t_{i}=1}>0$ as follows:

(11) $$L_{13}=\frac{N_{x_{i}\in \Omega }}{N}\sum_{x_{i}\in D_{T=1}\cap \Omega }\delta_{i}\max(0,{\hat{y}}_{i}^{T=1-t_{i}}-\gamma_{i}^{t_{i}=1})$$

(iv) $\;t_{i}=1$, $\delta_{i}=0$ and $\Delta_{Y_{i}}>0$.

In this case, there is $Y_{i}^{T=1}>\gamma_{i}^{t_{i}=1}$ because the survival time is censored, and therefore $Y_{i}^{T=1}$ has a lower bound $\gamma_{i}^{t_{i}=1}$ but $Y_{i}^{T=0}$ is counterfactual. However, it is obvious that $Y_{i}^{T=1}>\gamma_{i}^{t_{i}=1}$ and $Y_{i}^{T=1}-Y_{i}^{T=0}>0$ cannot yield any bound information for $Y_{i}^{T=0}$ (and further for ${\hat{y}}_{i}^{T=0})$ based on $\gamma_{i}^{t_{i}=1}$. So, in this case, the prior knowledge does not offer additional information for the counterfactual model training.

2. Patients with prior knowledge $\Delta_{Y_{i}}=0$ $(x_{i}\in \Gamma$)
  (i) $t_{i}=1$ or 0, $\delta_{i}=1$ and $\Delta_{Y_{i}}=0$.

In this case, the survival time can be observed, so similar to (1).(i) and (1).(iii), for $t_{i}=1$ or 0, $Y_{i}^{T=t_{i}}$ has the ground truth $\gamma_{i}^{t_{i}}$, i.e.,$\;Y_{i}^{T=t_{i}}=\gamma_{i}^{t_{i}}$, with $\gamma_{i}^{t_{i}}\;$equaling the true survival time, but $Y_{i}^{T=1-t_{i}}$ is counterfactual. Then, the prior knowledge $Y_{i}^{T=1}=Y_{i}^{T=0}$ for $x_{i}\in \Gamma$ is equivalent to $Y_{i}^{T=1-t_{i}}=Y_{i}^{T=t_{i}}=\gamma_{i}^{t_{i}}$ for $t_{i}=1$ or 0. Hence, $\gamma_{i}^{t_{i}}$ can serve as the label of the counterfactual predicted survival time ${\hat{y}}_{i}^{T=1-t_{i}}$, and the loss item can be defined as follows:

(12) $$L_{21}=\frac{N_{x_{i}\in \Gamma }}{N}\sum_{j=0,1}^{}\sum_{x_{i}\in D_{T=j}\cap \Gamma }\frac{1}{N_{x_{i}\in D_{T=j}\cap \Gamma ,T=j}}\delta_{i}\left|\gamma_{i}^{t_{i}}-{\hat{y}}_{i}^{T=1-t_{i}}\right|$$

where $N_{x_{i}\in \Gamma }$ denotes the number of patients who belong to Γ and $N_{x_{i}\in D_{T=j\cap^{\;}}\Gamma ,T=j}$ denotes the number of patients who belong to the intersection of $D_{T=j}$ (j=0 or 1) and Γ.

(ii) $t_{i}=1$ or 0, $\delta_{i}=0$ and $\Delta_{Y_{i}}=0$.

In this case, the survival time cannot be observed, so similar to (1). (ii) and (1).(iv), there is $Y_{i}^{T=t_{i}}>\gamma_{i}^{t_{i}}$ for $t_{i}$=1 or 0, and $Y_{i}^{T=1-t_{i}}$ is counterfactual. Then, the prior knowledge $Y_{i}^{T=1}=Y_{i}^{T=0}$ for $x_{i}\in \Gamma$ is equivalent to $Y_{i}^{T=1-t_{i}}=Y_{i}^{T=t_{i}}>\gamma_{i}^{t_{i}}$. Hence, we have a constraint ${\hat{y}}_{i}^{T=1-t_{i}}>\gamma_{i}^{t_{i}}$ on the counterfactual predicted survival time ${\hat{y}}_{i}^{T=1-t_{i}}$ with $\gamma_{i}^{t_{i}}$ as the lower bound, and we can define the loss item as:

(13) $$\;L_{22}=\frac{N_{x_{i}\in \Gamma }}{N}\sum_{j=0,1}^{}\sum_{x_{i}\in D_{T=j}\cap \Gamma }\frac{1}{N_{x_{i}\in D_{T=j}\cap \Gamma ,T=j}}\left[(1-\delta_{i})\max\left(0,\gamma_{i}^{t_{i}}-{\hat{y}}_{i}^{T=1-t_{i}}\right)\right]$$

whose minimization will penalize $\gamma_{i}^{t_{i}=0}-{\hat{y}}_{i}^{T=1-t_{i}}>0$.

### 4.5. Training Algorithm for CDNEPK

The final loss item for the counterfactual prediction ${\hat{y}}_{i}^{T=1-t_{i}}$ can be defined as the summation of (9)–(13), i.e.,

(14) $$\begin{array}{l} L_{\mathrm{CP}}=\frac{N_{x_{i}\in \Omega }}{N}\{\sum_{x_{i}\in \Omega \cap D_{T=0}}\delta_{i}\max(0,\gamma_{i}^{t_{i}=0}-{\hat{y}}_{i}^{T=1-t_{i}})+\sum_{x_{i}\in \Omega \cap D_{T=0}}\left(1-\delta_{i})\max(0,\gamma_{i}^{t_{i}=0}-{\hat{y}}_{i}^{T=1-t_{i}}\right)\\ \;\;\;\;\;+\sum_{x_{i}\in \Omega \cap D_{T=1}}\delta_{i}\max(0,{\hat{y}}_{i}^{T=1-t_{i}}-\gamma_{i}^{t_{i}=1})\}\\ +\frac{N_{x_{i}\in \Gamma }}{N}\sum_{j=0,1}^{}\sum_{x_{i}\in D_{T=j}\cap \Gamma }\left\{\frac{1}{N_{x_{i}\in D_{T=j}\cap \Gamma ,T=j}}\left\{\delta_{i}\left|\gamma_{i}^{t_{i}}-{\hat{y}}_{i}^{T=1-t_{i}}\right|+\left[(1-\delta_{i})\max\left(0,\gamma_{i}^{t_{i}}-{\hat{y}}_{i}^{T=1-t_{i}}\right)\right]\right\}\right\} \end{array}$$

and the loss function for CDNEPK is finally defined as:

(15) $$\underset{\begin{array}{c} \phi ,\psi \\ g_{T=1,}h_{T=1,}u_{T=1}\\ g_{T=0,}h_{T=0,}u_{T=0} \end{array}}{\min}\;L_{CDNEPK}=\min\left\{L_{CSA-Dragon}+L_{\mathrm{CP}}\right\}$$

where *L_CSA-Dragon_* has been defined in (3). By now, all of the four problems mentioned in Section 1, i.e., (i) balancing the confounders, (ii) handling the censored data, (iii) avoiding balancing the non-confounders, and (iv) taking advantage of prior knowledge have been properly considered in CDNEPK.

The training algorithm of CDNEPK is summarized as the following.

**Remark 2.**  *It is worth noting that Algorithm 1 can also be used for the* *training procedure of* *CSA–Dragonnet proposed in* Section 3 *just by replacing the loss fun* *ction in line four of Algorithm 1 (i.e.,* *Formula (15))* *as the loss fun* *ction of CSA–Dragonnet (i.e.,* *Formula (3)).*

**Algorithm 1:** Training algorithm of CDNEPK.**Input:** Dataset *D_all_*, weighting factors *α*,*β*, iteration time *c*_1_, batch number *c*_2_, batch size *b*, learning rate *r*, initial weights of network *W*;**Output:** Trained CDNEPK model1:    **for**
*i* = 1 to *c*_1_
**do**2:          
$\mathrm{Resort}\;\mathrm{and}\;\mathrm{divide}\;\mathrm{the}\;\mathrm{dataset}\;D_{all}\;\mathrm{into}\;c_{2}\;\mathrm{batches}\;{\left\{D^{j}\right\}}_{j=1}^{c_{2}}$
3:    
$\mathrm{for}\;j\;=1\;\mathrm{to}\;c_{2}\;\mathrm{do}$
4: Calculate loss function of *j*th batch *D^j^* according to Formula (15):


$$L_{CDNE\mathrm{PK}}\left(D^{j}\right)=L_{CSA-D\mathrm{ragon}}\left(D^{j}\right)+L_{\mathrm{CP}}\left(D^{j}\right)$$

5: Update *W* by descending its gradient

$$W\leftarrow W-\;r\cdot \nabla_{W}L_{\mathrm{CDNEPK}}\left(D^{j}\right)$$

6:          **end for**7:    **end for**

## 5. Experiments Based on Semi-Synthetic Data

### 5.1. Data Generating and Experiment Setup

As mentioned in Section 1, the results of RCTs or RCSs can offer valuable information about the ITE to counterfactual learning. In the following, two examples are given to illustrate it in detail.

Based on an ACTG dataset which is given by [31] and contains 2139 HIV patients who received either the treatment of “monotherapy with Zidovudine” or the treatment of “Diadanosine with combination therapy”, [4] proposes the following scheme for generating the semi-synthetic dataset $D_{all}=\left\{\left(\gamma_{i},x_{i},\delta_{i},t_{i}\right),\;i=1,2,\ldots ,N\right\}$ [4].

(16) $$\begin{array}{l} x_{i}=ACTG\;covariates\;of\;\mathrm{patients}\;i\\ P(t_{i}|x_{i})=1/d_{1}\times (d_{2}+\mathrm{sigmoid}(x_{i,AGE}-{\;\bar{x}}_{i,AGE}+\lambda x_{i,CD40}-\mu {\;\bar{x}}_{i,CD40}))\\ Y_{i}^{T=1}=\frac{1}{\kappa_{T=1}}\log\left[1-\frac{\kappa_{T=1}\log(Z=z_{i})}{\chi_{T=1}\exp(x_{i}{}^{T}\eta_{T=1})}\right]\\ Y_{i}^{T=0}=\frac{1}{\kappa_{T=0}}\log\left[1-\frac{\kappa_{T=0}\log(Z=z_{i})}{\chi_{T=0}\exp(x_{i}{}^{T}\eta_{T=0})}\right]\\ y_{i}^{t_{i}}=Y_{i}^{{T=t}_{i}}\\ \log y_{\mathrm{cen},i}∼Normal(\mu_{c},\sigma_{c}^{2})\\ \gamma_{i}^{t_{i}}=\min\left(y_{i}^{t_{i}},y_{\mathrm{cen},i}\right)\delta_{i}=1if{\;y}_{i}^{t_{i}}<y_{\mathrm{cen},i},\;\mathrm{else}\;\delta_{i}=0\; \end{array}$$

where the treatment $t_{i}$ is simulated via a logistic model; ${\bar{x}}_{i,AGE}$ and ${\bar{x}}_{i,CD40}$ are the average values of AGE and CD40; the potential outcomes $Y_{i}^{T=1}$ and $Y_{i}^{T=0}$ are simulated via the Gompertz-COX model; the survival time $y_{i}^{t_{i}}$ equals its corresponding $Y_{i}^{T=t_{i}}$; the censored time $y_{cen,i}$ is assumed to follow a lognormal distribution; and the observed time $\gamma_{i}$ is the minimum of the survival time $y_{i}^{t_{i}}$ and the censored time $y_{cen,i}$. $\delta_{i}=1\left(0\right)\;$indicates that survival time is (is not) censored, which is determined by comparing $y_{cen,i}$ and $y_{i}^{t_{i}}$ in the simulation, e.g., if $y_{cen,i}$ is longer than $y_{i}^{t_{i}}$, $\delta_{i}$ is set as 1 [4]. $\Lambda =\{d_{1}$, $d_{2}$, λ, μ, $\kappa_{T=1}$, $\kappa_{T=0}$, $\eta_{T=1}$, $\eta_{T=0}$, $\chi_{T=1}$, $\chi_{T=0}$, $\mu_{c}$, $\sigma_{c}^{2}\}$ contains the parameters of the simulation scheme. The Individual Treatment Effect $\Delta_{Y_{i}}$ can be acquired by $\Delta_{Y_{i}}=Y_{i}^{T=1}-Y_{i}^{T=0}$. It is worth mentioning that (16) can output both of the two potential outcomes $\;Y_{i}^{T=1}$ and $Y_{i}^{T=0}$, which is impossible in the real world and can be used to evaluate the performance of a counterfactual learning method which treats $y_{i}^{T=t_{i}}$ as counterfactual and unobservable.

In this section, to generate semi-synthetic data with simulated prior knowledge, we divided all the patients’ baselines covered by the ACTG dataset [31] into four cases, i.e.,

(17) $$\left\{ \begin{array}{l} \Theta_{1}=\left\{x_{i}|\begin{array}{l} 0.5I(x_{i,\;AGE}>30)+I(x_{i,\;AGE}>40)+0.5I(x_{i,\;\mathrm{CD}80}>500)+I(x_{i,\;\mathrm{CD}80}>800)+I(x_{i,\;Z30}=1)\\ +I(x_{i,\;\mathrm{RACE}}=1)+I(x_{i,\;\mathrm{GENDER}}=1)+I(x_{i,\;\mathrm{STE}}=1)\in \left\{5,\;5.5,\;6\right\} \end{array}\right\}\\ \Theta_{2}=\left\{x_{i}|\begin{array}{l} I(x_{i,\;AGE}>30)+I(x_{i,\;\mathrm{CD}80}>500)+I(x_{i,\;Z30}>0.5)+I(x_{i,\;\mathrm{RACE}}=1)+I(x_{i,\;\mathrm{GENDER}}=1)\\ +I(x_{i,\;\mathrm{STE}}=1)\in \left\{6\right\} \end{array}\right\}\\ \Theta_{3}=\left\{x_{i}|\begin{array}{l} 0.5I(x_{i,\;AGE}>30)+I(x_{i,\;AGE}>40)+0.5I(x_{i,\;\mathrm{CD}80}>500)+I(x_{i,\;\mathrm{CD}80}>800)+I(x_{i,\;Z30}=1)\\ +I(x_{i,\;\mathrm{RACE}}=1)+I(x_{i,\;\mathrm{GENDER}}=1)+I(x_{i,\;\mathrm{STE}}=1)\in \left\{0,\;0.5,\;1,\;1.5,\;2\right\} \end{array}\right\}\\ \Theta_{4}=\left\{x_{i}|x_{i}\notin \Theta_{1}\cup \Theta_{2}\cup \Theta_{3}\right\} \end{array}\right.$$

and then by setting the parameters in Λ of (16) properly, generated four different datasets satisfying different conditions, respectively, i.e., $D_{1}=\left\{x_{i}|x_{i}\in \Theta_{1}\;\mathrm{and}\;\Delta_{Y_{i}}>0\right\}$, $D_{2}=\left\{x_{i}|x_{i}\in \Theta_{2}\;\mathrm{and}\;\Delta_{Y_{i}}>0\right\}$, $D_{3}=\left\{x_{i}|x_{i}\in \Theta_{3}\;\mathrm{and}\;\Delta_{Y_{i}}=0\right\}$, and $D_{4}=\{x_{i}|x_{i}\in \Theta_{4}\;\mathrm{and}\;\Delta_{Y_{i}}\;\mathrm{has}\;\mathrm{wide}\;\mathrm{distribution}\}$. The final semi-synthetic dataset was obtained by $D_{all}=\cup_{i=1}^{4}D_{i}$. Through properly selecting the parameters in Λ, among the 2139 patients in $D_{all}$, there were 417 patients belonging to $D_{1}$ or $D_{2}$, 668 patients belonging to $D_{3}$, and 1054 patients belonging to $D_{4}$.

From the viewpoint of evaluating an ITE estimation method based on the dataset $D_{all}$, although $\Delta_{Y_{i}}$ was generated by the simulation and was known, we treated $\Delta_{Y_{i}}\;$as counterfactual (not observable) but assumed that there was the prior knowledge $\Delta_{Y_{i}}>0$ or $\Delta_{Y_{i}}=0$ for part of the patients, i.e., there were $\Delta_{Y_{i}}$ $=Y_{i}^{T=1}-Y_{i}^{T=0}>0$ if $x_{i}\in \Omega$ and $\Delta_{Y_{i}}$ $=Y_{i}^{T=1}-Y_{i}^{T=0}=0\;$if $x_{i}\in \Gamma$ with a high certainty, where $\Omega =\Theta_{1}\cup \Theta_{2}$ and $\Gamma =\Theta_{3}$, and we had no prior knowledge for patients not belonging to Ω or Γ, among which $\Delta_{Y_{i}}$ may have randomly varied from negative to positive.

In the experiment, the dataset was randomly divided into the training set, validation set, and test set with a ratio of 70%:15%:15%. As in CSA [4], the FCNs ϕ, $g_{T=1},\;g_{T=0}$, $u_{T=1}$, $u_{T=0}$ used in CDNEPK and CSA–Dragonnet were two-layer MLPs of 100 hidden units, and the FCNs $h_{T=1}$,$\;h_{T=0}$ used in CDNEPK and CSA–Dragonnet were one-layer MLPs. In addition, all the hidden units in ϕ, $g_{T=1},\;g_{T=0}$ were characterized by batch normalization and the dropout probability of 𝑝 = 0.2 on all layers. As in Dragonnet [10], the FCN ψ used in CDNEPK and CSA–Dragonnet was a one-layer MLP. Weighting factors α, β in (3) were set as 1000,100, respectively, which were selected by cross-validation. The iteration time c was set as 80 and the batch size was set as 850. An Adam optimizer was used with the learning rate r =$\;3\times 10^{-3}$.

### 5.2. Experimental Results

We compared our proposed CDNEPK and CSA–Dragonnet with the following methods: (i) CSA [4]; (ii) the accelerate failure time (AFT) model with Weibull distributions [12]; (iii) the random survival forest (RSF) model [13]; and (iv) the COX proportional hazard model [11]. Among them, CSA was introduced in the preliminary, whose settings for the FCNs were identical to those in CDNEPK and CSA–Dragonnet in the simulation. Instead of applying balance representation like the three methods mentioned above, the AFT, RSF, and COX models took the treatment vector as a covariate directly, which led to the limited ability to handle selection bias.

In the experiments, we adopted the PEHE (precision in the estimation of a heterogeneous effect) and the absolute error of the ATE (average treatment effect), which are widely used for assessing the Individual Treatment Effect error [4], and are defined as following, respectively [4]:

(18) $$\varepsilon_{PEHE}=\sqrt{\frac{1}{N}{\sum_{i\leq N}\left(\Delta_{Y_{i}}-{\hat{\Delta }}_{Y_{i}}\right)}^{2}}$$

(19) $$\varepsilon_{ATE}=\left|\frac{1}{N}\sum_{i\leq N}{\hat{\Delta }}_{Y_{i}}-\frac{1}{N}\sum_{i\leq N}\Delta_{Y_{i}}\right|$$

It is worth noting that $\varepsilon_{\mathrm{PEHE}}$ and $\varepsilon_{\mathrm{ATE}}$ can only be calculated in simulation experiments where the ground truth $\Delta_{Y_{i}}$ is available and they cannot be calculated for real-world data where $\Delta_{Y_{i}}$ is counterfactual [4].

Table 2 presents the comparison results among COX, AFT, RSF, CSA, CSA–Dragonnet, and CDNEPK. It can be seen that the COX and AFT models had poorer performance since they adopted linear models and did not consider selection bias. For RSF, although it still suffered from selection bias, its ability to process nonlinear survival data led to the lower $\varepsilon_{\mathrm{PEHE}}$ and $\varepsilon_{\mathrm{ATE}}$ compared to COX and AFT.

CSA, as the baseline method of this paper, dealt with the nonlinearity and selection bias by representation learning and balancing the confounders. It had a significant enhancement compared to COX, AFT, and RSF. Compared to the basis of CSA, the proposed CSA–Dragonnet took the confounder identification into account and improved the performance on $\varepsilon_{\mathrm{PEHE}}$ and $\varepsilon_{\mathrm{ATE}}$. Furthermore, CDNEPK is proposed to cope with prior knowledge, which is superior to all other methods.

## 6. Real-World Experiment on Hepatocellular Carcinoma

As the third most fatal cancer for men in the world, Hepatocellular Carcinoma (HCC) has a high mortality rate for patients [32]. Although a hepatectomy is the most effective treatment for HCC, the mortality of some patients after a hepatectomy still remains high and how long a hepatectomy can prolong the survival time of HCC patients still remains controversial [33]. In this section, we utilized CDNEPK to estimate the Individual Treatment Effect for each patient.

The dataset used in this section included records of 1459 patients, which were retrospectively collected from three hospitals in China. Among the 1459 patients, 784 patients were treated with a hepatectomy and the other 675 patients were not treated with liver resection. Basic information, laboratory tests, and imaging tests were included in the patients’ records. The basic information included gender, age, and ECOG-PS score. The laboratory tests consisted of alpha-fetoprotein (AFP), blood tests (i.e., total bilirubin, alanine transaminase, aspartate aminotransferase, and alkaline phosphatase), and hepatitis tests (i.e., HBsAg, HBsAb, HBeAg, HBeAb, HBcAb, and HCVAb). The imaging tests contained tumor numbers, diameters, sites, distant metastasis, vascular invasion, and ascites. All of the above 21 clinical covariates of the baseline and whether a patient had a hepatectomy were included in our final analysis.

In Example 2 we mentioned that HCC patients with a single small tumor cannot benefit from a hepatectomy with a high probability. As for HCC patients in other cases, there are also RCTs or RCSs that focus on whether they could benefit from a hepatectomy. [34] summarizes the results as follows: (i) patients with a single tumor lower than 2 cm, no distant metastasis (DM), no vascular invasion (VI), and no ascites (AC) could not benefit from a hepatectomy significantly;(ii) patients with 2–3 tumors lower than 2 cm, no distant metastasis (DM), no vascular invasion (VI), and no ascites (AC) could not significantly extend survival time from a hepatectomy;(iii) patients with a single tumor between 5–10 cm, no distant metastasis (DM), no vascular invasion (VI), and no ascites (AC) could benefit from a hepatectomy significantly.

Similar to Example 2 of Section 4.1, the results of the RCTs and RCSs given in [34] can be expressed as the following prior knowledge. Let $Y_{i}^{T=1\left(0\right)}$ represent the potential survival time of patient i receiving a hepatectomy (not receiving a hepatectomy), and we divide all patients’ baselines covered by the HCC dataset [31] into four cases, i.e.,

(20) $$\{\begin{array}{l} D_{1}=\left\{x_{i}|x_{i}\in \Theta_{1}^{}\;\mathrm{and}\;\Delta_{Y_{{}_{i}}}=0\right\}\\ D_{2}=\left\{x_{i}|x_{i}\in \Theta_{2}^{}\;\mathrm{and}\;\Delta_{Y_{{}_{i}}}=0\right\}\\ D_{3}=\left\{x_{i}|x_{i}\in \Theta_{3}^{}\;\mathrm{and}\;\Delta_{Y_{{}_{i}}}>0\right\}\\ D_{4}=\left\{x_{i}|x_{i}\in \Theta_{4}^{}\;\mathrm{and}\;\Delta_{Y_{{}_{i}}}\;\mathrm{has}\;\mathrm{wide}\;\mathrm{distribution}\right\} \end{array}$$

where

(21) $$\left\{ \begin{array}{l} \Theta_{1}=\left\{x_{i}|I(x_{i,diameter}\leq 2)+I(x_{i,number}=1)+I(x_{i,DM}=0)+I(x_{i,VI}=0)+I(x_{i,AC}=0)\in \left\{4\right\}\right\}\\ \Theta_{2}=\left\{x_{i}|I(x_{i,diameter}\leq 2)+I(2\leq x_{i,number}\leq 3)+I(x_{i,DM}=0)+I(x_{i,VI}=0)+I(x_{i,AC}=0)\in \left\{4\right\}\right\}\\ \Theta_{3}=\left\{x_{i}|I(5\leq x_{i,diameter}\leq 10)+I(x_{i,number}=1)+I(x_{i,DM}=0)+I(x_{i,VI}=0)+I(x_{i,AC}=0)\in \left\{4\right\}\right\}\\ \Theta_{4}=\left\{x_{i}|x_{i}\notin \Theta_{1}\cup \Theta_{2}\cup \Theta_{3}\right\} \end{array}\right.$$

According to Formula (7), there is $\Gamma =\Theta_{1}\cup \Theta_{2}$ and $\Omega =\Theta_{3}$.In addition, for patients not belonging to Ω or Γ, we have no prior knowledge, among which $\Delta_{Y_{i}}$ may have a wide distribution.

In the experiment, we obtained a trained CDNEPK by using Algorithm 1 of Section 4.5 based on the data of the 1459 HCC patients, in which the settings of CDNEPK were identical to those in Section 5.

A direct usage of the obtained CDNEPK is giving the predicted ITE by ${\hat{\Delta }}_{Y_{new}}\left(x_{new}\right)$ for a new patient who has not received treatment yet, where ${\hat{\Delta }}_{Y_{new}}\left(x_{new}\right)$ denotes the output of CDNEPK with $x_{new}$ as the input. This kind of prediction may help a doctor or patient choose the proper treatment. However, the reason why we did not divide the dataset of the 1459 HCC patients into a training set and a test set to show the predicted ITEs for the patients in the test set and evaluate their prediction errors is that the ITE is counterfactual for a patient in the real word data, which means the ground truth data is unavailable for any patient.

In the following, we will show another usage of the obtained CDNEPK, i.e., analyzing the importance of each covariate on the ITE. Based on the obtained CDNEPK, we first calculate ${\hat{\Delta }}_{Y_{i}}$ for all of the 1459 patients based on their baselines, then build the relationship between the estimated ITE and the baseline by solving the following lasso regression problem:

(22) $$\underset{\;\varpi }{\min}\;\left\{\frac{1}{2N}\sum_{i=1}^{N}{\left({\hat{\Delta }}_{Y_{i}}-\varpi_{0}-\sum_{j=1}^{m}x_{ij}\varpi_{j}\right)}^{2}+\iota {\left\|\varpi \right\|}_{1}\right\}$$

where ϖ = $\left[\varpi_{1},\ldots \varpi_{m}\right]$ and ι are the regression coefficient vector and weighting factor, respectively. Formula (22) can be solved by the method in [20]. According to the idea of factor analysis [35], it is intuitive that the absolute values of the m regression coefficients, i.e., $\varpi_{1},\ldots \varpi_{m}$, can reflect the contributions of the m covariates of the baseline $x_{i}$ to the ITE, respectively; i.e., the greater $\left|\varpi_{j}\right|$ is, the greater the contribution the jth covariate has to the ITE. So, through cross-validation, we selected four covariates corresponding to the regression coefficients with the top four greatest absolute values as the key covariates which are most important to the ITE, i.e., tumor diameter, alpha fetoprotein, aspartate aminotransferase, and distant metastasis.

**Figure 4:**
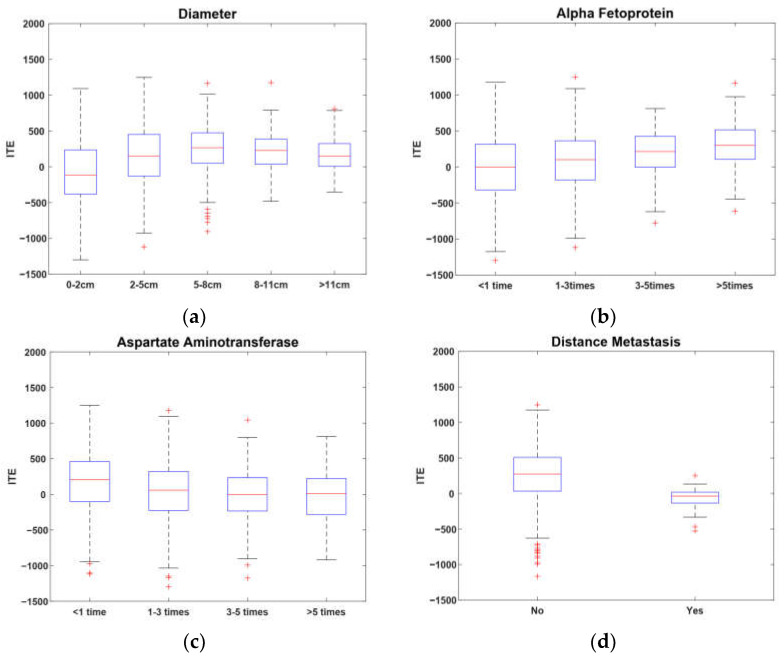
Key covariates of ITE.

In Figure 4, a box-plot is used to illustrate the relationships between the ITE and the four key covariates. It is apparent from Figure 4a that the ITE increased with the increase in tumor diameter when it was less than 8 cm. In contrast, when the diameter was less than 2 cm, the median $\Delta_{Y_{i}}$ was less than zero, which indicates that patients with numbers less than 2 cm may not benefit significantly from a hepatectomy. As a whole, patients with tumors between 5–8 cm could benefit the most from a hepatectomy. Figure 4b indicates that the ITE increased with the increase in alpha fetoprotein, while Figure 4c shows that the ITE decreased with the increase in aspartate aminotransferase. It can be inferred that the benefit of a hepatectomy is positively associated with liver function. Figure 4d shows that patients without distant metastasis had higher benefit ratios than those with distant metastasis in terms of the median and upper quartile. Thus, patients without distant metastasis have a high probability of benefiting from a hepatectomy.

The above example shows that, with CDNEPK, we can utilize observational historical data and prior knowledge to estimate the individual surgical benefit for HCC patients and can further analyze the influence of covariates on the trend of surgical benefits. The results can offer HCC surgeons quantitative information and valuable assistant treatment advice, which can never be obtained by RCT or RCS studies.

## 7. Conclusions

In this paper, we propose CSA–Dragonnet and CDNEPK to estimate the ITE on survival time from observational data. The key novelty of our methods is that we insert counterfactual prediction nets into CSA–Dragonnet and extract valuable bound information for the counterfactual prediction from the prior knowledge yielded by RCTs and RCS to guide the training of counterfactual outputs. Experiments based on semi-synthetic data and real-world data showed that CDNEPK had the best performance compared to existing methods and that it can provide auxiliary treatment advice for surgeons.

## Figures and Tables

**Table 1:** Child-Pugh score (CP score) [24,25].

Covariates	Conditions for Covariate Score = 1	Conditions for Covariate Score = 2	Conditions for Covariate Score = 3
hepatic encephalopathy grade	0	1, 2	3, 4
ascites grade	0	1	2, 3
total bilirubin (g/L)	>0 and <34	34~51	>51
albumin (g/L)	>35	28~35	>0 and <28
prothrombin time (s)	>0 and <4	4~6	>6

**Table 2:** Quantitative Results.

	$\varepsilon_{\mathrm{PEHE}}$	$\varepsilon_{\mathrm{ATE}}$
COX	375.33	144.65
AFT	342.71	180.08
RSF	292.78	127.29
CSA	291.49	80.34
CSA–Dragonnet	271.23	73.24
**CDNEPK**	**264.59**	**67.35**

## Data Availability

Not applicable.
